# Lung transplantation for patients with severe COVID-19

**DOI:** 10.1126/scitranslmed.abe4282

**Published:** 2020-11-30

**Authors:** Ankit Bharat, Melissa Querrey, Nikolay S. Markov, Samuel Kim, Chitaru Kurihara, Rafael Garza-Castillon, Adwaiy Manerikar, Ali Shilatifard, Rade Tomic, Yuliya Politanska, Hiam Abdala-Valencia, Anjana V. Yeldandi, Jon W. Lomasney, Alexander V. Misharin, G. R. Scott Budinger

**Affiliations:** 1 Division of Thoracic Surgery, Northwestern Memorial Hospital, Feinberg School of Medicine, Northwestern University, Chicago, IL 60611, USA.; 2 Division of Pulmonary and Critical Care Medicine, Northwestern Memorial Hospital, Feinberg School of Medicine, Northwestern University, Chicago, IL 60611, USA.; 3 Department of Biochemistry and Molecular Genetics, Northwestern Memorial Hospital, Feinberg School of Medicine, Northwestern University, Chicago, IL 60611, USA.; 4 Department of Pathology, Northwestern Memorial Hospital, Feinberg School of Medicine, Northwestern University, Chicago, IL 60611, USA.

## Abstract

Lung transplantation can potentially be a life-saving treatment for patients with nonresolving COVID-19–associated respiratory failure. Concerns limiting lung transplantation include recurrence of SARS-CoV-2 infection in the allograft, technical challenges imposed by viral-mediated injury to the native lung, and the potential risk for allograft infection by pathogens causing ventilator-associated pneumonia in the native lung. Additionally, the native lung might recover, resulting in long-term outcomes preferable to those of transplant. Here, we report the results of lung transplantation in three patients with nonresolving COVID-19–associated respiratory failure. We performed single-molecule fluorescence in situ hybridization (smFISH) to detect both positive and negative strands of SARS-CoV-2 RNA in explanted lung tissue from the three patients and in additional control lung tissue samples. We conducted extracellular matrix imaging and single-cell RNA sequencing on explanted lung tissue from the three patients who underwent transplantation and on warm postmortem lung biopsies from two patients who had died from COVID-19–associated pneumonia. Lungs from these five patients with prolonged COVID-19 disease were free of SARS-CoV-2 as detected by smFISH, but pathology showed extensive evidence of injury and fibrosis that resembled end-stage pulmonary fibrosis. Using machine learning, we compared single-cell RNA sequencing data from the lungs of patients with late-stage COVID-19 to that from the lungs of patients with pulmonary fibrosis and identified similarities in gene expression across cell lineages. Our findings suggest that some patients with severe COVID-19 develop fibrotic lung disease for which lung transplantation is their only option for survival.

## A surgical solution for severe COVID-19

Despite optimal medical therapy, some patients with severe COVID-19 develop irreversible lung injury. In these patients who cannot be weaned from mechanical ventilation or extracorporeal life support, lung transplantation may be the only life-saving option. Bharat *et al*. now report the results of lung transplantation in three patients who had COVID-19–associated respiratory failure. SARS-CoV-2 RNA could not be detected in the explanted lungs of these patients, but fibrotic pathology and transcriptional changes resembling those of lungs from patients with pulmonary fibrosis were observed.

## Teaser

Some patients with severe COVID-19 develop end-stage pulmonary fibrosis for which lung transplantation may be the only treatment.

## INTRODUCTION

As of 20 November 2020, over 12 million people have been diagnosed with coronavirus disease 2019 (COVID-19) in the United States and nearly 260,000 have died (https://coronavirus.jhu.edu), with greater than 4.6 million active cases. Globally, 57.9 million cases have been reported with about 1.4 million deaths. In addition, there are greater than 16 million active cases worldwide, of which 102,303 remain in serious or critical condition (https://www.worldometers.info). In many patients, SARS-CoV-2 infection can progress to severe respiratory failure and acute respiratory distress syndrome (ARDS) requiring mechanical ventilation (*1*, *2*). The reported mortality of patients with COVID-19 requiring mechanical ventilation is between 20 and 40%, despite optimized supportive care (*3*, *4*).

Lung transplantation is a life-saving treatment for a variety of end-stage lung diseases (*5*). Annually, more than 2700 lung transplants are performed in the United States with a 1-year survival of over 90% and a 3-year survival of over 75% (www.srtr.org). Several concerns limit the use of lung transplantation as a therapy for patients with severe ARDS secondary to COVID-19. First, there is a concern that SARS-CoV-2 or superinfecting pathogens associated with viral pneumonia in the native lung might recur in the allograft. Second, severe vascular and pleural damage secondary to SARS-CoV-2 infection might create technical barriers to transplant, increase the time that tissues are ischemic, and worsen outcomes. Third, the severe deconditioning associated with prolonged mechanical ventilation, sedation, and neuromuscular blockade might complicate recovery after transplant. Fourth, there is uncertainty as to whether the lung can repair itself after severe SARS-CoV-2–associated pneumonia; effective lung repair would result in long-term outcomes better than lung transplantation.

Here, we successfully performed lung transplantation in three consecutive patients with SARS-CoV-2 infection–associated severe pneumonia, who required prolonged mechanical ventilation and extracorporeal membrane oxygenation (ECMO) support and in whom recovery was determined to be unlikely. We examined explanted native lung tissue from the transplant recipients as well as postmortem lung tissue from patients with COVID-19 who had died of ARDS. Histopathology, single-molecule fluorescence in situ hybridization (smFISH) for detecting SARS-CoV-2 RNA, tissue clearing to image extracellular matrix organization, and single-cell RNA sequencing (RNA-seq) were conducted on the lung tissue samples. Lung histology and extracellular matrix imaging revealed evidence of severe fibrosis in the explanted lungs. SARS-CoV-2 viral transcripts were not detected in the lung explants using smFISH, and there was no evidence of recurrent SARS-CoV-2 infection in the allograft. Machine learning–based analysis of single-cell RNA-seq data revealed similarities between lung tissue from patients with COVID-19–induced respiratory failure and published RNA-seq datasets derived from lung tissue from patients with pulmonary fibrosis, suggestive of common pathways leading to irreversible lung tissue damage and fibrosis. We found that lung disease after severe and prolonged SARS-CoV-2 infection–associated ARDS shared pathological and molecular features with pulmonary fibrosis requiring lung transplantation, suggesting that lung transplantation may be the only option for survival in these patients.

## RESULTS

### Clinical profiles of study participants

The clinical profiles of three patients with COVID-19 (cases 1, 2, and 3), who underwent bilateral lung transplantation, are presented below and in Table 1. In addition to these three cases, lung tissue samples from other patients with lung disease were also analyzed (table S1). These included postmortem lung biopsies from two patients who had died from severe COVID-19 after prolonged mechanical ventilation and ECMO support (referred to as PMB1 and PMB2); a biopsy from a donor lung from an unrelated transplant (referred to as donor 1); a biopsy of the donor lung from the first lung transplant (referred to as donor 2); native lungs explanted from two patients who underwent lung transplantation for idiopathic pulmonary fibrosis (referred to as IPF1 and IPF2); a control autopsy lung specimen from a patient who died from pulmonary embolism (PE) without marked lung pathology (referred to as PE control); and a lung biopsy sample from a patient who died from SARS-CoV-2–associated pneumonia while receiving palliative care (referred to as palliative COVID-19).

**Table 1:** Clinical characteristics of lung transplant recipients. Continuous data are shown as means ± SD. BMI, body mass index; VA ECMO, veno-arterial extracorporeal membrane oxygenation; ICU, intensive care unit.

**Variable**	**Patients with COVID-19 (3)**
Age, years	44.3 ± 13.9
Female	1 (33.3%)
BMI, kg/m^2^	25.2 ± 4.5
Operating time (hours)	9.5 ± 1.0
Intraoperative blood transfusion	
packed red blood cells	10.6 ± 4.1
fresh frozen plasma	5.3 ± 2.4
platelets	2.6 ± 1.2
Intraoperative VA ECMO use	3 (100%)
Intraoperative VA ECMO time (hours)	2.8 ± 0.3
Ischemic time (hours)	5.1 ± 0.1
ICU stay (days)	13.3 ± 7.0
Posttransplant ventilator (days)	12.6 ± 8.3
Pleural drainage (days)	20.3 ± 4.4

### Clinical profile of case 1

**Fig. 1:**
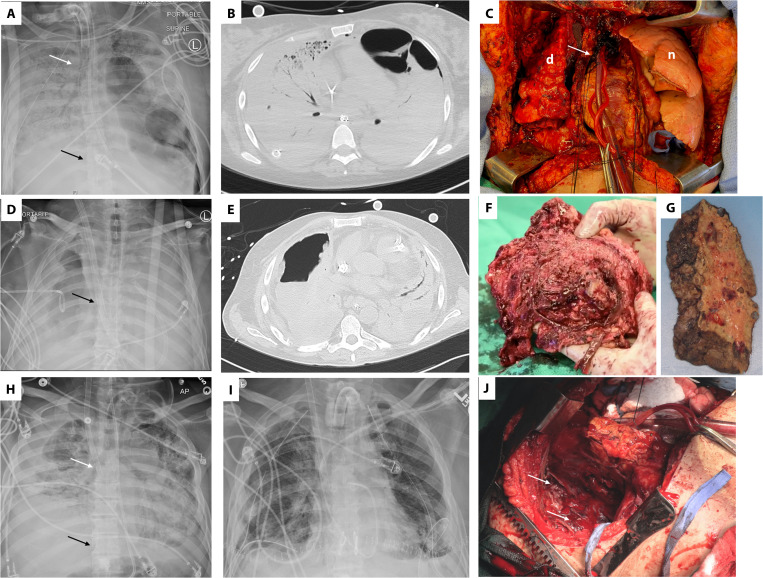
Radiographic and intraoperative findings in lung transplant recipients with severe COVID-19. (**A** to **C**) Radiographic and intraoperative findings for case 1 with severe COVID-19 who underwent lung transplantation. (A) Pretransplant chest radiograph (day 38 after onset of ARDS) for case 1, revealing opacification of the right lung and a left lower lobe necrotic cavity attributed to pneumonia caused by *Serratia marcascens*. A tube thoracostomy was required to treat right spontaneous hemothorax and peumothoraces. In addition, the image shows ECMO cannulas (inflow cannula carrying oxygenated blood to the patient is indicated by the white arrow, and outflow cannula carrying deoxygenated blood from the patient to the ECMO machine is indicated by the black arrow). (B) Severe ARDS and lower lung lobe necrosis in case 1 were confirmed by cross-sectional computed tomography imaging. (C) Shown is an intraoperative image revealing contrasting features between the diseased native right lung (d) and the newly transplanted left lung (n). The photograph was taken immediately after the transplant of the left lung and before proceeding onto right lung transplantation. The pericardial sac (white arrow) containing the heart was opened to gain access to the aorta and place the outflow cannula of the venoarterial ECMO. (**D** to **F**) Radiographic and intraoperative findings for case 2 with severe COVID-19 who underwent lung transplantation. (D) Pretransplant chest radiograph (at day 98 after onset of ARDS) for case 2 revealing bilateral lung opacifications and a necrotic cavity in the right lung attributed to pneumonia caused by *Pseudomonas aeruginosa*. A chest tube to treat bronchopleural fistula is visible. The dual lumen ECMO cannula is indicated by the black arrow. (E) Computed tomography imaging indicates severe ARDS and development of a necrotic cavity in the right lung. (F) Freshly explanted right lung of case 2 with extensive pleural inflammation and loss of identifiable anatomical planes. (**G**) Formalin-fixed explanted right lung of case 1 demonstrating the development of lung cavities. (**H** to **J**) Radiographic and intraoperative findings for case 3 with severe COVID-19 who underwent lung transplantation. (H) Pretransplant chest radiograph (day 86 after onset of severe ARDS) for case 3, revealing extensive consolidation (lack of air) with right fibrothorax. In addition, the image shows ECMO cannulas (inflow cannula carrying oxygenated blood to the patient is indicated by the white arrow, and the outflow cannula carrying deoxygenated blood from the patient to the ECMO machine is indicated by the black arrow). (I) Posttransplant day 1 chest radiograph for case 3 demonstrating the expected appearance of new lung allografts after bilateral lung transplantation. (J) Intraoperative photograph after implantation of the left lung and explantation of the right lung revealing the right hemithorax. Diffuse pleuritis with severe thickening of parietal pleura and neoangiogenesis (white arrows) were noted as in the other two cases.

A 28-year-old Latina female with neuromyelitis optica, who was being treated with rituxamab and mycophenolate mofetil, presented with 2 weeks of poor appetite, gastrointestinal discomfort, fevers, cough, dyspnea on exertion, and pleuritic chest pain and was diagnosed with COVID-19. Upon presentation, she exhibited severe hypoxemia that was refractory to oxygen therapy and underwent emergency intubation. Nasopharyngeal swab and bronchoalveolar lavage (BAL) were positive by polymerase chain reaction (PCR) for SARS-CoV-2 RNA. She received mechanical ventilation according to ARDSNet guidelines (*6*) using a higher positive end-expiratory pressure (PEEP)/lower fraction of inspired oxygen (FiO_2_) strategy. Her partial pressure of oxygen (PaO_2_)/FiO_2_ ratio was <150 persistently, and she was ventilated prone for a total of three 16-hour periods in the first week of her illness (*7*). Bronchoscopic sampling of the alveolar space was performed after intubation and when clinical coinfection or superinfection with respiratory pathogens was suspected. The results of quantitative cultures and multiplex PCR detection for respiratory pathogens (BioFire FilmArray Respiratory 2 Panel) were used to manage antibiotic treatment over the course of her hospitalization. Despite these interventions, the patient’s blood oxygenation concentrations continued to decrease, and she was placed on veno-venous ECMO. Her clinical course was complicated by a right-sided pneumothorax requiring multiple pleural tubes and the development of pneumonia caused by *Serratia marcescens* with left lower lung necrosis (Fig. 1, A and B). Later in her disease course, she developed elevated pulmonary arterial pressure (71/49 mmHg), and echocardiography showed moderate right ventricular dysfunction with severe tricuspid regurgitation and congestive hepatopathy. Systemic anticoagulation was initiated but was complicated by a hemothorax and a liver capsular bleed necessitating emergent exploratory laparotomy. Throughout her medical course, she received broad-spectrum and pathogen-directed antibiotics, remdesivir, hydroxychloroquine, tocilizumab, and convalescent plasma. Her lung compliance, gas exchange, chest radiographs, and chest tomogram showed worsening or no improvement over the course of her stay in the intensive care unit (ICU). Beginning 6 weeks from the onset of mechanical ventilation, PCR testing for SARS-CoV-2 in BAL fluid samples was repeatedly negative. Weaning the patient off of the ECMO was attempted over the course of several weeks with no success, and she was listed for lung transplantation. Before lung transplantation, several attempts were made to wean the patient off of sedation, but they were associated with severe hypoxemia and hemodynamic collapse. Computed tomography imaging of the head and electroencephalograms did not demonstrate irreversible brain injury but were suggestive of delirium. After obtaining consent by the designated medical power of attorney, the patient was listed for lung transplantation. Two days after listing, she received a bilateral lung transplant using central venoarterial ECMO. Severe dense vascular adhesions were noted in both of the patient’s native lungs with severe distortion of hilar planes and reactive lymphadenopathy (Fig. 1C). After lung transplantation, she received maintenance immunosuppression with tacrolimus, mycophenolate mofetil, and prednisone. After the transplant, her sedation was stopped, and she awakened without respiratory distress or hemodynamic instability. In the ensuing 2 weeks, she was separated from the veno-venous ECMO as well as from mechanical ventilation and was discharged for inpatient rehabilitation 4 weeks after transplantation. Her neurocognitive status and muscular strength improved rapidly after the lung transplantation. About 5 months after transplantation, the patient demonstrated oxygen saturations above 98% when breathing room air and reported independence in activities of daily living.

### Clinical profile of case 2

A 62-year-old male with hypertension was placed on veno-venous ECMO for severe COVID-19–induced ARDS before transfer to our institution. During his clinical course, he received remdesivir, convalescent plasma, antibiotics, and dexamethasone. His pretransplant hospital stay was complicated by recurrent pneumonia caused by *Pseudomonas aeruginosa*, hemothorax, and empyema requiring thoracotomy and lung decortication (Fig. 1, D to F). Lung compliance and blood oxygenation failed to recover, and he could not be weaned from the veno-venous ECMO. Beginning 4 weeks after hospitalization, repeated bronchoscopic sampling of bronchoalveolar fluid was negative for SARS-CoV-2 RNA by PCR. Before the transplant, the patient was awake and participated in physical rehabilitation in the ICU. After 100 days on veno-venous ECMO support, there were no signs of lung recovery; he was listed for transplant and underwent bilateral lung transplantation 3 days later. The intraoperative findings were similar to the first patient, but increased complexity was encountered due to liquefactive necrosis from secondary *Pseudomonas* pneumonia and the prior lung decortication procedure. There was complete loss of normal mediastinal tissue planes and extensive pleuritis in the explanted native lungs (Fig. 1F), similar to the explanted lungs of the first patient (Fig. 1G). Four months after lung transplantation, the patient had oxygen saturations over 97% when breathing room air and reported independence in activities of daily living.

### Clinical profile of case 3

A 43-year-old man with medically controlled type 2 diabetes mellitus required mechanical ventilation and veno-venous ECMO 3 and 6 days, respectively, after presenting with acute hypoxemic respiratory failure due to severe COVID-19 to an outside institution. His medical course was complicated by an asystolic cardiac arrest on day 23, heparin-induced thrombocytopenia, a left frontal lobe infarct of the cerebral cortex, *S. marcescens*–mediated pneumonia and bacteremia, acute kidney injury, a left hemothorax requiring thoracotomy and lung decortication, a right pneumothorax requiring tube thoracostomy, hypernatremia associated with seizures, malnutrition, and a tracheostomy. Despite these complications, he was weaned off sedation and was able to participate in physical rehabilitation in the ICU before being considered for lung transplantation. During his clinical course, he received remdesivir, convalescent plasma, pathogen-directed antibiotics, as well as steroids. From 4 weeks after intubation, repeated bronchoscopic sampling of bronchoalveolar fluid was negative for SARS-CoV-2 RNA by PCR. However, because of the failure of lung recovery with increasing clinical signs of progressive lung fibrosis, he was transferred to our center. He was evaluated for lung transplant and listed. Six days later, he underwent bilateral lung transplantation using central venoarterial ECMO about 90 days after the initial development of ARDS (Fig. 1, H and I). Given his recent medical history of heparin allergy and heparin-induced thrombocytopenia, we performed the lung transplantation without the use of anticoagulation (*8*–*11*). The patient was found to have severe pulmonary hypertension with moderate right ventricular dysfunction. Increased surgical complexity including severe pleural and mediastinal adhesions with lymphadenopathy obscuring the mediastinal structures in the native lungs was encountered, likely secondary to the prior thoracotomy and hemothorax and ventilator-associated pneumonia (Fig. 1J). About 3 months after lung transplantation, the patient had oxygen saturations over 95% when breathing room air and continued to improve regarding neurocognitive status and muscular strength at an inpatient rehabilitation facility.

### Histological evaluation of lung tissue biopsies

**Fig. 2:**
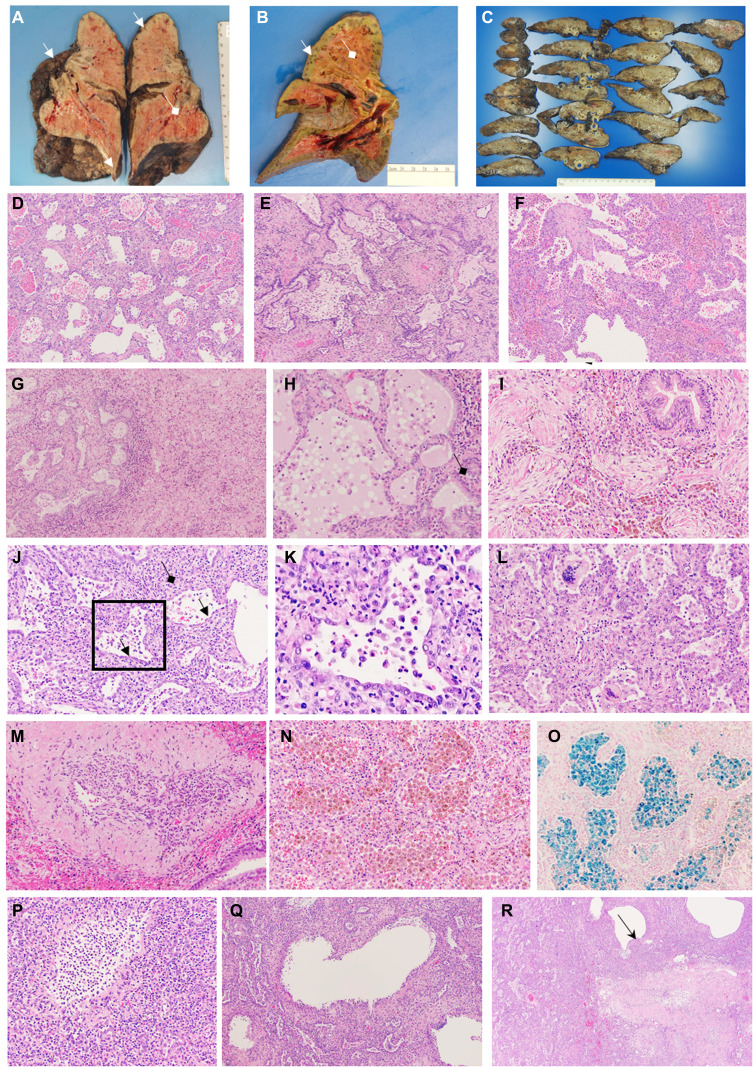
Common histological features of lung explants. Shown are common histological features of lung explants from three patients with severe COVID-19 who underwent lung transplantation. (**A** to **C**) Gross pathology images of explanted lungs from case 1 (A), case 2 (B), and case 3 (C). Cystic structures are evident on the lung surface and in the lung parenchyma (white arrows) along with diffuse fibrosis. Purulent secretions in the airways suggestive of bronchopneumonia are indicated by white diamond arrows. (**D** to **R**) Remaining images show sections of the explanted lung from cases 1, 2, and 3 stained with hematoxylin and eosin, except for image (O), which was stained with Prussian blue to detect iron deposition. (D) Image shows lung alveoli in the explanted lung from case 1 demonstrating hemorrhage, interstitial fibrosis, and prominent reactive pneumocytes (100×). (E) Bronchiolitis and bronchiolar fibrosis with microscopic honeycombing were observed for the explanted lung from case 2 (200×). (F) Organizing areas of alveolar hemorrhage are visible on this image of explanted lung from case 3 (100×). (G) Microscopic honeycombing adjacent to an area of more dense fibrosis with interstitial expansion and inflammatory infiltrates is visible on this image of explanted lung from case 2 (100×). (H) Bronchiolitis and fibrosis (black diamond arrow) are visible on this image of explanted lung from case 2 (200×). (I) An area of organizing pneumonia showing whorls of fibroblasts in the airways and interstitial expansion is observed on this image of explanted lung from case 2 (200×). (J) Interstitial fibrosis and bronchiolar fibrosis (black arrows) are visible in this image of explanted lung from case 2 (100×). (K) Enlarged inset from (J) reveals a bronchiole surrounded by interstitial fibrosis and cuboidal epithelia. (L) Area of interstitial fibrosis and microscopic honeycombing with multinucleated giant cells in the airspaces is visible on this image of explanted lung from case 1 (200×). (M) A medium-size blood vessel with an organizing thrombus and recannulation can be observed in this image of explanted lung from case 2 (100×). (N) Interstitial inflammation and expansion with alveoli filled with pigmented macrophages are visible in this image of explanted lung from case 1 (100×). (O) Staining for iron revealed alveolar macrophages laden with hemosiderin (blue) in this image of explanted lung from case 1 (100×). (P) This image of explanted lung from case 1 shows formation of cystic airspaces with neutrophilic inflammatory infiltrates (200×). (Q) This image of explanted lung from case 1 reveals cystic airspaces lined by histiocytes and hyperplastic epithelia (100×), and (R) this image reveals a mature cyst (black arrow) near an area of airway fibrosis (40×).

At the time of transplantation, the lungs from the three patients were edematous and heavy [right lung weight: 595.7 g (case 1), 476.3 g (case 2), and 345 g (case 3); left lung weight: 496.2 g (case 1), 548.2 g (case 2), and 353.9 g (case 3)]. In case 1, the external surface of the lung explant had a nodular appearance and dense fibrous adhesions between the lobes (Fig. 2A). In all three patients, there were dense pleural adhesions, and the first patient had visible pleural blebs and cysts (Fig. 2, A to C). In the three lung explants examined, cavities with necrosis were observed. Postoperative cultures confirmed the presence of bacterial pathogens in these cavities, which were detected preoperatively by PCR in BAL fluid.

Lung tissue obtained from cases 1, 2, and 3 at the time of transplantation and from warm autopsy material obtained within 1 hour of death had several shared features (Fig. 2 and fig. S1) that were consistent with other autopsy series from patients who had died from COVID-19 (*12*). These features included regions of diffuse alveolar hemorrhage, as well as acute bronchopneumonia from secondary bacterial infection (Fig. 2, D to F). In other areas, the lungs showed various stages of a pattern consistent with an acute interstitial pneumonitis including acute neutrophilic infiltrates within the interstitium and alveolar spaces, interstitial expansion by fibrosis, bronchiolization of alveoli, and areas of microscopic honeycomb changes (Fig. 2, G and H). In addition, organizing pneumonia showing whorls of fibroblasts in the airways and interstitial expansion was observed (Fig. 2I). Rare microthrombi were observed, some with recanalization (Fig. 2J). Case 2 developed lung infarction and necrosis secondary to larger thrombi. Alveolar macrophages within the airspaces stained positive for iron, confirming the presence of alveolar hemorrhage (Fig. 2, K and L). Sections from the lung explant of case 1 showed multiple cystic structures in various stages of formation. Early cyst formation consisted of acute bronchilolitis with intraluminal neutrophils (Fig. 2M), then chronic inflammation with histiocytic cells and giant cells lining the bronchioles (Fig. 2N and fig. S1I). More mature cysts were devoid of inflammatory cells, and some were associated with fibrosis (Fig. 2, O to R). Whereas the changes in cyst formation that we observed might reflect changes associated with prolonged mechanical ventilation, similar structures were observed in another patient who died from COVID-19 after deciding to forgo mechanical ventilation. Some of these cystic changes with histiocytic and giant cell responses were reminiscent of pneumatoceles, possibly from peripheral airway destruction as a result of viral or bacterial infection.

### Analysis of explanted and warm autopsy lung tissue

**Fig. 3:**
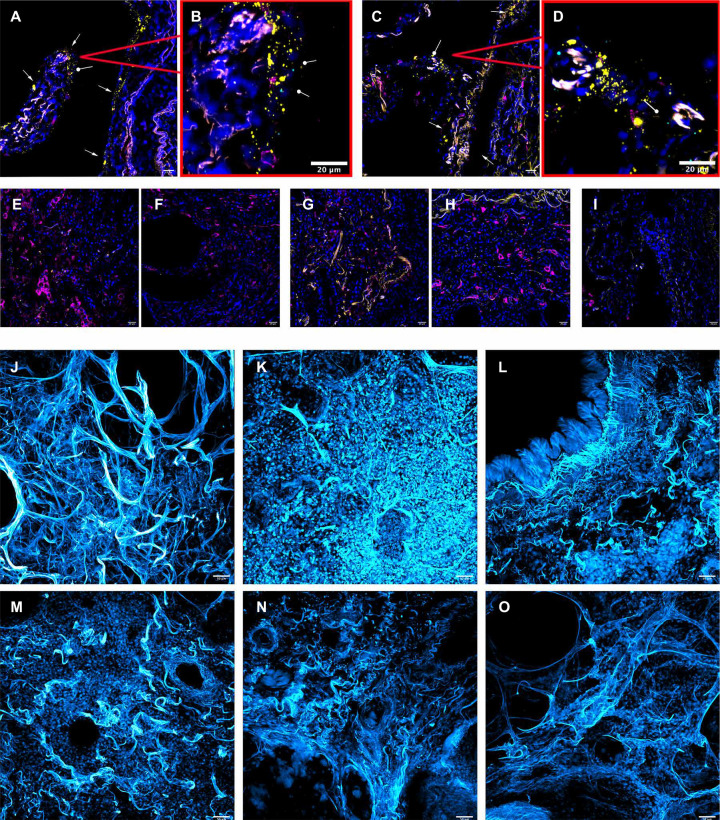
smFISH and matrix imaging in cleared lung sections from patients with severe COVID-19. (**A** to **D**) RNAScope and immunohistochemistry of lung autopsy tissue from a patient who declined interventions for COVID-19–induced respiratory failure (palliative COVID-19). Nuclear staining (blue), positive strand SARS-CoV-2 RNA (yellow), negative strand SARS-CoV-2 RNA (cyan), and CD206 (magenta). Positive strand SARS-CoV-2 RNA (yellow) was detected in cells with morphological features suggestive of epithelial cells (white arrows); negative strand SARS-CoV-2 RNA was also detected (cyan; circular arrow). (**E** and **F**) RNAScope images of the explanted lung from case 1 who underwent lung transplantation. There is yellow autofluorescence but no staining for SARS-CoV-2 positive or negative strand RNA in either left (E) or right (F) explanted lung. (**G** and **H**) RNAScope images of the explanted lung of case 2 who underwent lung transplantation, showing no evidence of SARS-CoV-2 RNA. (**I**) RNAScope image of a postmortem lung biopsy from a patient who died of COVID-19 (PMB1), showing no evidence of SARS-CoV-2 RNA. (**J** to **O**) Cleared lung tissue allowing visualization of the collagen structure and matrix of lung tissue (cyan); ×20 magnification. (J) Normal lung from a patient who died of pulmonary embolism. (K) An explanted lung from case 1 who underwent lung transplantation. (L and M) Shown are postmortem lung biopsies from two patients who died of COVID-19 (PMB1 and PMB2). (N and O) Shown are lung explants from two patients with idiopathic pulmonary fibrosis (IPF1 and IPF2) who underwent lung transplantation.

We examined the explanted lung tissue of the three lung transplant patients (cases 1, 2, and 3) obtained 40 and 100 days after the initiation of mechanical ventilation. Before the transplant, BAL fluid samples were tested repeatedly for the presence of SARS-CoV-2 by PCR testing and were found to be negative. SARS-CoV-2 is a positive RNA strand virus, and during replication, a negative strand is transiently formed to serve as a template for the positive strand (*13*). The presence of both positive and negative RNA strands in a cell is suggestive of ongoing viral replication. Accordingly, we performed smFISH using the RNAscope assay with probes designed to detect positive and negative RNA strands of SARS-CoV-2 in sections of explanted lung tissue. Lung tissue obtained from a patient who had forgone therapy for respiratory failure secondary to COVID-19 (palliative COVID-19, control) showed many positive and few negative strands of SARS-CoV-2 RNA (Fig. 3A to D). In contrast, we were unable to detect virus using this technology in the lung explants from cases 1 and 2 who underwent lung transplantation (Fig. 3, E to H) or in postmortem lung tissue from patients with COVID-19 who had undergone prolonged extracorporeal life support (PMB1; Fig. 3I and table S1). Postoperative PCR performed on BAL fluid samples confirmed a lack of SARS-CoV-2 recurrence after lung transplantation.

Pulmonary fibrosis is characterized by the development of aberrant and disorganized collagen deposition in the alveolar regions of the lung. We sought to compare the three-dimensional (3D) matrix organization of end-stage lung tissue from patients with SARS-CoV-2–associated pneumonia and patients undergoing lung transplantation for idiopathic pulmonary fibrosis (IPF). We adapted our protocols for tissue clearing using a method called SHIELD (stabilization under harsh conditions via intramolecular epoxide linkages to prevent degradation) to generate and image the 3D matrix organization of lung tissue. A postmortem lung sample from a patient who had died from PE with relatively normal histology (PE control; Fig. 3J and movie S1) showed bands of matrix surrounding terminal airways with wispy lines defining the airways and airspaces that were devoid of cells or matrix. Images from explanted lung tissue from case 1 showed a complete absence of matrix organization with punctate islands of cells surrounding fibrotic airway regions (Fig. 3K). A similar pattern was observed in lung tissue from two patients who had died from severe COVID-19–associated pneumonia after a similar duration of mechanical ventilation (patients PMB1 and PMB2; Fig. 3, L and M, and movie S1). This disorganized matrix pattern was also observed in lung explant sections from two patients with IPF who underwent lung transplantation (patients IPF1 and IPF2; Fig. 3, N and O, and movie S1).

### Transcriptional analysis of lung explant and warm autopsy samples

**Fig. 4:**
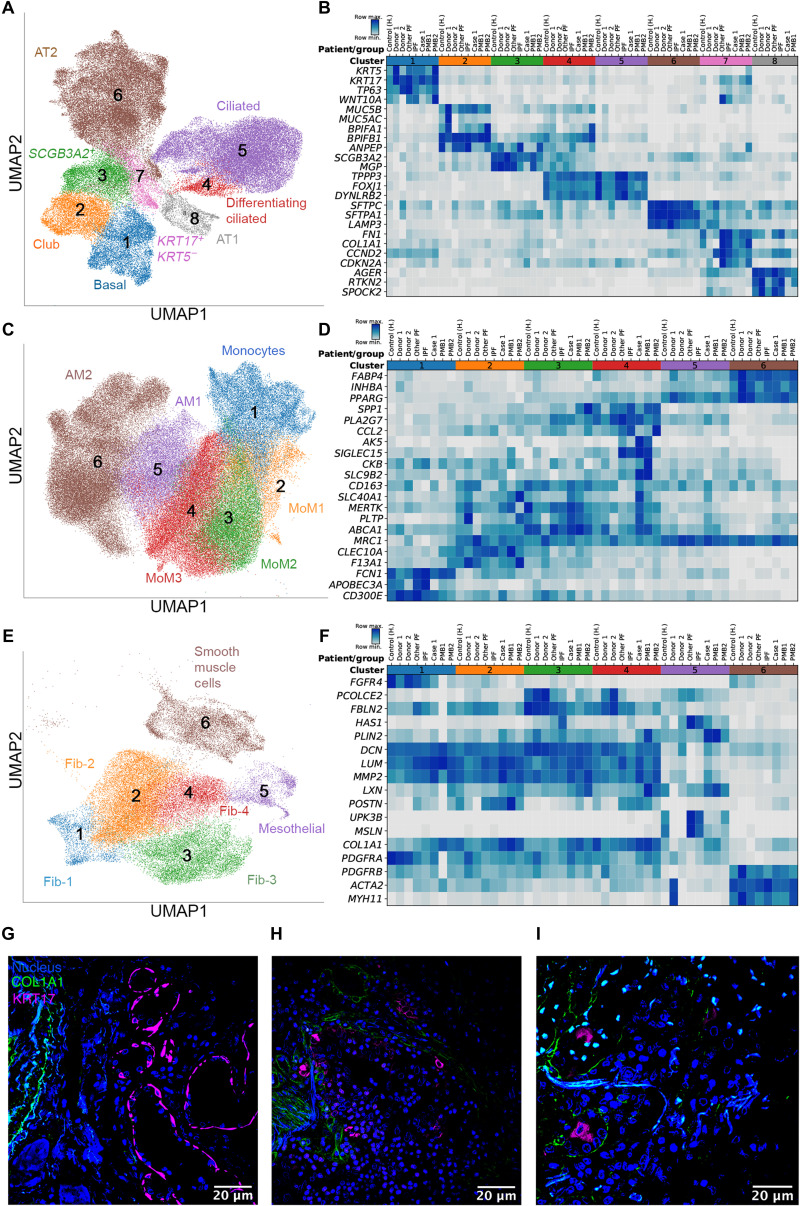
Single-cell RNA sequencing of lung tissue from patients with severe COVID-19. (**A**, **C**, and **E**) Uniform Manifold Approximation and Projection (UMAP) plots showing individual populations of epithelial cells (A), macrophages (C), and mesenchymal cells (E). (**B**, **D**, and **F**) Heatmaps illustrating expression of select marker genes in epithelial cells (B), macrophages (D), and mesenchymal cells (F). Gene expression for the pulmonary fibrosis dataset of Habermann *et al.* (*16*) is shown as an average per condition; gene expression for the end-stage COVID-19 dataset is shown per individual patient. Labels on heatmaps (B, D, and F) correspond to the following samples: Control (H), healthy controls; IPF, idiopathic pulmonary fibrosis samples; Other PF, samples from patients with other forms of pulmonary fibrosis, all from the Habermann *et al.* dataset. Donor 1, Donor 2, control donor lungs; Case 1, lung transplant case 1; PMB1, PMB2, postmortem lung biopsies from two patients with COVID-19, all from the end-stage COVID-19 dataset. (**G**) Immunofluorescence microscopy revealed KRT17 staining (magenta) of flat epithelial cells resembling alveolar type 1 cells in nonfibrotic lung tissue from a patient who died of COVID-19 (palliative COVID-19). (**H** and **I**) Immunofluorescence microscopy revealed KRT17 staining (magenta) of distal explanted lung tissue from a patient with COVID-19 undergoing lung transplantation (H) and lung tissue from a patient (PMB1) who died from late-stage severe COVID-19 (I). Normal lung architecture is lacking, and solitary KRT17-positive cells (magenta) can be observed close to COL1A1-positive cells (green). Scale bars, 20 um.

Our histological and extracellular matrix analysis demonstrated many similarities between the lung tissue from patients with end-stage COVID-19 and patients with end-stage pulmonary fibrosis. To test whether COVID-19–associated fibrosis correlated with specific immune, epithelial, and mesenchymal cell populations known to be associated with development of pulmonary fibrosis (*14*–*17*), we performed single-cell RNA-seq on lung explant tissue from the first lung transplant patient (case 1), postmortem lung samples from patients PMB1 and PMB2, and two normal donor lung samples (donor 1 and donor 2). Consistent with our smFISH results, we did not detect SARS-CoV-2 RNA transcripts in lung tissue samples from patients PMB1 and PMB2 with end-stage COVID-19 disease. We compared our single-cell RNA-seq data to that for lung tissue from patients with pulmonary fibrosis using a well-annotated atlas generated by Habermann *et al*. (*16*) and a transfer learning approach called scArches (*18*). From our single-cell RNA-seq data, we identified cell populations (epithelial cells, macrophages, and mesenchymal cells) in postmortem lung tissue from patients PMB1 and PBM2 that were very similar to those reported in lung tissue from patients with pulmonary fibrosis (Fig. 4, A, C, and E, and fig. S2, A and B). In contrast, our single-cell RNA-seq data for normal donor lung tissue identified cell populations that mapped onto corresponding cell populations in normal lungs in the reference dataset (Fig. 4, A, C, and E) (*16*).

The alignment of cell populations in lung tissue from patients with end-stage COVID-19 and patients with end-stage pulmonary fibrosis prompted us to explore similarities and differences in these cell populations between these two disease groups. We began with epithelial cells, as an emerging literature suggests that pulmonary fibrosis begins with disordered alveolar epithelial repair. Specifically, as alveolar type 2 cells differentiate into alveolar type 1 cells, a transitional cell population of cells characterized by increased expression of *Krt8* arises in animal models of pulmonary fibrosis (*19*–*22*). An analogous population of cells characterized by the expression of *TP63*, *KRT5*, *KRT17*, *LAMB3, LAMC2*, *VIM*, *CHD2*, *FN1*, *COL1A1*, *TNC*, and *HMGA2* and several senescence markers (*CDKN1A*, *CDKN2A*, *CCND1*, *CCND2*, *MDM2*, and *SERPINE1*) accumulates during pulmonary fibrosis in humans. These cells have been suggested to contribute to disease pathogenesis through expression of *TGFB1*, *ITGAV*, and *ITGB6* (the *KRT5*^−^*KRT17^+^* epithelial cell cluster in the Habermann *et al*. dataset) (*16*, *17*). A similar epithelial cell population with similar gene expression patterns was observed in lung tissue from case 1 and from patients PMB1 and PMB2; this cell population was absent from donor lung tissue (donor 1 and donor 2) (Fig. 4, A and B, fig. S2C, and table S3). We validated the presence of this epithelial cell population in the lung explant parenchyma of two patients with COVID-19 (cases 1 and 2) who underwent lung transplantation (Fig. 4, G to I). Warm autopsy lung tissue from a COVID-19 patient with early disease, who did not receive mechanical ventilation and had lung parenchyma free of fibrotic changes, was used as a control (palliative COVID-19; Fig. 4G). In normal healthy lung, expression of *KRT17* is restricted to basal, club, ciliated, and alveolar type 1 cells (*14*, *16*). We observed flat KRT17-positive cells resembling alveolar type 1 cells in the distal lung of the patient with early COVID-19 disease who died (palliative COVID-19; Fig. 4G). In contrast, explanted lung tissue from cases 1 and 2 who underwent lung transplantation contained KRT17-positive cells that were localized near COL1A1-positive cells and in some regions appeared to line the cyst-like structures revealed by standard histology (Fig. 4, H and I). Direct comparison of differential gene expression in basal cells (cluster 1) or *KRT17*^+^*KRT5*^−^ cells (cluster 7) in lung tissue from patients with COVID-19 or patients with pulmonary fibrosis did not identify genes uniquely present in one condition, highlighting similarities between these two diseases (table S4).

Lung epithelial injury results in the continuous recruitment of monocyte-derived alveolar macrophages to the alveolar space that is required for the development of fibrosis in animal models (*23*). We observed abundant macrophages in the airways and alveolar spaces of lung explants from cases 1 and 2 (Fig. 2). Human profibrotic monocyte-derived alveolar macrophages are characterized by expression of *SPP1*, *ILRN*, *MMP9*, *CHI3L1,* and *PLA2G7* and reduced expression of *FABP4*, *INHBA*, and *PPARG,* which mark tissue-resident homeostatic alveolar macrophages present in the normal lung (*14*–*17*). Tissue-resident alveolar macrophages were present in healthy donor lungs and in a reference dataset (clusters 5 and 6) but were nearly absent in lung samples from patients with COVID-19 (Fig. 4, C and D, fig. S2D, and table S4). In contrast, macrophages with a transcriptional profile matching profibrotic monocyte-derived alveolar macrophages (cluster 4) were observed in all three patients with end-stage COVID-19 showing pulmonary fibrosis and in patients with other fibrotic lung diseases; these cells were not observed in healthy donor lungs (fig. S2D). Direct comparison of differential gene expression in cluster 4 macrophages between patients with pulmonary fibrosis and patients with COVID-19 demonstrated similarities between these two diseases (table S4). Our analysis also identified a small group of cells in the lung samples from patient PMB1 and case 1 that expressed genes including *AK5*, *SIGLEC15*, *CKB*, and *SLC9B2*, suggesting that these cells could have been osteoclasts in areas of lung necrosis and calcification (Fig. 4D and fig. S2F). Another population of macrophages (cluster 3) was enriched in the lung explant from case 1, but not in postmortem biopsies from patients PMB1 and PMB2. These macrophages expressed genes involved in iron (*SLC40A1* and *CD163*) and lipid (*MERTK*, *PLTP*, and *ABCA1*) metabolism, and cell motility and immune signaling (*MARCKS*, *TLR2*, and *CCL20*), potentially reflecting ongoing inflammation (Fig. 4, C and D, and fig. S2D).

Profibrotic macrophages are thought to activate and stimulate the proliferation of fibroblasts by forming self-sustaining cellular circuits maintained by growth factor release from fibroblasts and macrophages (*24*, *25*). The scArches projection identified several clusters of mesenchymal cells including subsets of fibroblasts, myofibroblasts, smooth muscle cells, and mesothelial cells in normal donor lung tissue and lung tissue from patients with end-stage COVID-19 (Fig. 4, E and F, and table S3). Fibroblast clusters 1 and 2 (which matched myofibroblasts in the reference dataset) were overrepresented in lung samples from patients with COVID-19 (fig. S2E). Cluster 2 cells showed increased expression of *POSTN* and *COL1A1* and other extracellular matrix genes implicated in pulmonary fibrosis (Fig. 4F and table S3). Gene expression profiles for fibroblasts in lung tissue from patients with pulmonary fibrosis and patients with severe COVID-19 were similar (table S5).

## DISCUSSION

We performed successful lung transplantation in three patients with respiratory failure that was secondary to SARS-CoV-2–induced pneumonia. All patients had severe disease and required ECMO to maintain adequate oxygenation. Furthermore, these three patients had similar complications from SARS-CoV-2 infection, which necessitated prolonged supportive ICU care. Complications included markedly reduced lung compliance, repeated episodes of ventilator-associated pneumonia with increasingly resistant nosocomial pathogens, pneumothoraces requiring repeated tube thoracostomy, and bleeding into both the pleural space and airways. In addition, the pathology of lung explants from these patients showed extensive and severe damage with marked similarities. These included diffuse alveolar damage, areas of bronchopneumonia with necrosis, extensive pleural inflammation, rare microthrombi, alveolar hemorrhage, and the accumulation of hemosiderin-labeled alveolar macrophages and occasional giant cells in the lung. In addition, the explanted lungs of the first two patients (cases 1 and 2) showed extensive bilateral cysts that were visible on lung sections by microscopy. Microscopic examination revealed that these cysts were extensive, variable in size, and were lined by dysplastic cuboidal or sometimes squamous epithelium. In the interstitium surrounding these cysts, the normal alveolar architecture was largely lost with extensive interstitial thickening and fibrosis. Histological features of both acute and end-stage lung fibrosis in patients with severe COVID-19 may be the result of ongoing acute lung injury from SARS-CoV-2–induced pneumonia combined with complicating factors including nosocomial pneumonias, ventilator-induced lung injury, and thrombosis. Nevertheless, our pathological findings suggested that the damage and destruction to the lungs were irreversible, and lung transplantation was the only viable treatment option for these three patients.

Given that the duration of SARS-CoV-2 infection in the lungs of patients with severe disease is uncertain, we were concerned about ongoing SARS-CoV-2 infection at the time of lung transplantation and potential reinfection of the allograft, particularly in our first patient (case 1) who had received immunosuppression with rituximab and mycophenolate before her diagnosis with COVID-19. Accordingly, we performed repeated bronchoscopic sampling of multiple lung regions before transplant and tested samples by PCR. Reassuringly, we did not detect viral transcripts either by single-cell RNA-seq or by smFISH, suggesting that bronchoscopic sampling is a clinically useful method to exclude SARS-CoV-2 infection before consideration for lung transplant. Our data are consistent with recent studies suggesting that it is rare to detect replicating virus more than 10 days after infection with SARS-CoV-2 (*26*–*28*).

We treated the three patients who underwent lung transplantation for COVID-19 with a typical three-drug immunosuppression regimen including calcineurin inhibitors, an anti-metabolite, and steroids. They were also administered solumedrol as well as basiliximab before reperfusion as part of our induction immunosuppression protocol for all lung transplant patients. In addition, they received antimicrobial drugs directed toward the pathogens isolated from the native lungs before transplantation. All patients underwent repeat bronchoscopy after lung transplantation to rule out recurrence of infection with SARS-CoV-2 and bacterial pathogens. The consideration of lung transplantation for these three patients required certain deviations from the standard process. First, we did not have the opportunity to discuss the option of lung transplantation with the first patient given the inability to wean her off sedation. Hence, we relied on the medical power of attorney for decision-making. Second, these patients developed multiple nosocomial complications, neuromuscular deconditioning, and malnutrition due to their complicated medical course, which may be an impediment to transplantation in patients with long-standing end-stage lung disease. Whereas these factors are relative contraindications for lung transplantation, we reasoned that the normal functional status of the patients before their SARS-CoV-2–induced pneumonia would reduce the impact of these factors on recovery after lung transplantation. This is partly supported at least anecdotally by their outcomes to date. These three patients had single irreversible organ (lung) failure at the time of lung transplantation, which increased the likelihood of favorable posttransplant outcomes. Last, given the low likelihood of malignancy in these patients based on medical history, we omitted certain investigations directed toward cancer screening such as colonoscopy and mammography in these patients. Whereas longer-term follow-up in larger cohorts is needed, our experience supports the safety and feasibility of lung transplantation in carefully selected patients as a life-saving treatment despite a complex pretransplant medical course.

An emerging literature suggests a model for pulmonary fibrosis in which injury, often virally mediated, to the alveolar epithelium triggers the recruitment of monocyte-derived alveolar macrophages to the alveolar space (*23*). These macrophages function to seal the wound, in the process forming reciprocal circuits with fibroblasts in which macrophages produce factors stimulating fibroblast proliferation and matrix production, such as platelet-derived growth factor A (PDGFA) or sphingosine 1-phospate, while fibroblasts produce macrophage colony-stimulating factor (M-CSF) that maintains monocyte-derived alveolar macrophages in the lung alveolar niche (*24*). Meanwhile, alveolar type 2 cells and local alveolar epithelial progenitor cells differentiate into type 1 cells in response to incompletely understood signals (*20*, *29*–*31*). Failed injury repair allows macrophage-fibroblast circuits to persist, eventually filling the interstitium with matrix proteins and fibroblasts. With single-cell RNA-seq, we observed similarities between the end stages of pulmonary fibrosis and COVID-19–mediated pneumonia. Specifically, we observed the emergence of an abnormal population of basaloid-like epithelial cells expressing Keratin-17 that have been observed lining fibroblastic foci in patients with IPF, as well as profibrotic macrophages and myofibroblasts (*14*, *16*, *17*). Furthermore, both patients with IPF and those with end-stage COVID-19 pneumonia showed an increase in disorganized matrix deposition in the lungs. However, it is likely that the pathogenesis of pulmonary fibrosis in these patients is multifactorial with contributions from the cytopathic effects of the virus, inflammation, trauma related to the ventilator (barotrauma), and secondary bacterial pneumonia. Nevertheless, collectively, our data suggest that some patients with severe COVID-19 develop an irreversible fibrotic lung disease for which transplantation is likely their only option for survival (*32*).

We offer some recommendations based on our experience. First, we propose bilateral rather than single lung transplantation for patients with severe COVID-19 as all of our native lung explant samples included cavitary areas of bronchopneumonia and all of our recipients developed secondary pulmonary hypertension. Second, lung transplant should only be considered when sufficient time has elapsed to exclude possible spontaneous lung recovery, that is, typically after 4 to 6 weeks of mechanical ventilation, in the absence of severe complications necessitating earlier transplant. If validated, the identification of KRT17^+^KRT5^−^ basaloid cells or profibrotic alveolar macrophages in bronchoscopic biopsies may assist in identifying patients with irreversible lung fibrosis in the future. Third, the patients should be involved in the transplant decision whenever possible, recognizing that, in a small group of patients who require sedation for hemodynamic and respiratory stability, the consent to proceed with transplantation can be made through a reliable medical power of attorney, aligned with the patient’s health goals. Fourth, we required two negative PCR tests of bronchoalveolar fluid or nasopharyngeal swabs in nonintubated patients before their listing for lung transplant to ensure clearance of the virus (*26*–*28*). Fifth, we recognize that patients with severe COVID-19 have a shorter period of preoperative morbidity and may recover with intensive postoperative rehabilitation; hence, we encourage but do not require pretransplant rehabilitation. Last, some patients with severe COVID-19 have multiorgan failure. Whether multiorgan transplant for these patients is safe or feasible will require additional consideration.

Whereas we believe that our study supports the consideration of lung transplantation for patients who have developed irreversible lung disease due to COVID-19, more studies are needed to determine the long-term outcomes of lung transplantation in these patients. In addition, although our machine learning–based transcriptomics analysis supports the use of KRT17^+^KRT5^−^ basaloid cells or profibrotic alveolar macrophages in bronchoscopic biopsies as markers of irreversible lung disease in patients with severe COVID-19, further clinical studies are necessary to validate these findings.

## MATERIALS AND METHODS

### Study design

The current study included the first three consecutive patients undergoing lung transplantation for severe COVID-19. In addition, lung biopsies were collected from patients who died from severe COVID-19 or other lung diseases. Patient demographic and clinical data were collected prospectively in our lung transplant database. For the purposes of the study, a retrospective analysis was performed using the database. This study was approved by the Institutional Review Board of Northwestern University (STU00212120, STU00213177, STU00212511, and STU00212579). For inclusion in this study, patients or their designated medical power of attorney provided informed consent.

### Clinical characteristics of patients

The three patients with COVID-19 who underwent lung transplantation are described in Results and Table 1.

#### Postmortem biopsy patient 1 (PMB1)

A 54-year-old man with medically controlled hyperlipidemia presented with 5 days of dyspnea, cough, and diarrhea. A nasopharyngeal swab was positive for SARS-CoV-2 infection. He was intubated for acute hypoxemic respiratory failure. Ventilator management was guided by ARDSNet guidelines, and the patient underwent three rounds of proning. However, he developed worsening hypoxemia and hypercapnia, necessitating initiation of veno-venous ECMO. Throughout the ECMO support, he underwent verticalization therapy to improve lung recruitment. His medical course spanning 29 days was complicated with acute renal failure, spontaneous hemothorax, pulmonary hemorrhage, and ventilator-associated pneumonia. He first tested negative for SARS-CoV-2 in bronchoalveolar fluid samples on day 27 after symptom onset. He received hydroxychloroquine, remdesivir, and sarilumab. However, he progressed to multiorgan dysfunction and care was withdrawn, after which he expired within 40 min. Within 1 hour of death, warm autopsy of the left lung was performed. The tissue was processed for single-cell RNA-seq and SHIELD tissue clearing.

#### Postmortem biopsy patient 2 (PMB2)

A 57-year-old woman with no known medical conditions presented with dyspnea and was initially treated with a 1-week course of steroids and hydroxychloroquine. She was intubated for acute hypoxemic respiratory failure. She was managed with prone ventilation based on ARDSNet guidelines. After intubation, she received remdesivir but she continued to worsen requiring veno-venous ECMO. She received broad-spectrum antibiotics and systemic anticoagulation throughout her clinical course. She first tested negative for SARS-CoV-2 in bronchoalveolar fluid samples 31 days after admission. The medical course was complicated by multiorgan multipressor shock. Forty-eight days after admission, medical care was redirected to comfort care, and the patient expired within 10 min. Within 1 hour of expiration, the left lung was biopsied and processed for single-cell RNA-seq and SHIELD tissue clearing.

#### Palliative COVID-19 patient

An 81-year-old woman with end-stage renal disease and cirrhosis was admitted for a fever of 38.5°C. She had a positive nasopharyngeal swab for SARS-CoV-2. The patient developed increased O_2_ requirements and was subsequently transferred to the COVID-19 ICU. In the ICU, the patient developed hypotension, and after discussion with the clinical team, the patient’s family elected to focus on comfort care. The patient died 8 days after admission. A lung biopsy was taken at autopsy for smFISH to detect SARS-CoV-2 RNA.

### ICU management for lung transplant patients

Lung transplant case 2 received most of his care at a referring hospital where the details of his management were not known. At Northwestern University, case 2 and the other two patients (cases 1 and 3) were managed according to a standardized care plan for patients with COVID-19 that is similar to care for patients with ARDS. Specifically, all patients received mechanical ventilation according to ARDSNet criteria (low tidal volume, high PEEP) (*6*). Patients received prone ventilation when the PaO_2_/FiO_2_ ratio was <150; this was repeated for 16 hours daily until criteria were no longer met (*7*). Patients underwent BAL when pneumonia was suspected, and antibiotic therapy was guided by quantitative cultures or the results of multiplex PCR analysis of bronchoalveolar fluid samples (BioFire FilmArray Respiratory Viral Panel 2).

### Lung biopsy tissue fixation and thick slicing

Human lung biopsies collected during native lung explant or during postmortem biopsy were sliced to 5-mm thickness and placed in 4% paraformaldehyde solution for 72 hours at room temperature. Samples were then exchanged to 70% ethanol and stored in 4°C until use. Biopsies were also sent to the clinical pathology laboratories where they were fixed, paraffin embedded, and stained according to standard clinical protocols.

Biopsy samples were sliced to 100-μm thickness using a VT1200S Leica vibratome at 3.0-mm amplitude and 0.7 mm/s speed. For healthy lung biopsies, 100-μm-thick slices were made at 3.0-mm amplitude and 0.35 to 0.4 mm/s speed with ice in the holding chambers. Slices were stored in a tissue culture plate in 1× phosphate-buffered saline (PBS) with 0.2% sodium azide; the tops and sides were sealed in parafilm. Plates were stored in 4°C until use.

### RNAscope on paraffin-embedded lung slices

RNAscope Multiplex V2 manual assay from ACDbio was performed on paraffin-embedded 5-μm slices of lung tissue using mild digest times according to the manufacturer’s instructions as we have described (*14*). Probes used were RNAscope Probe-V-nCoV2019-S-C3 (catalog number 848561) with Akoya Bio Opal Dye 520 using the 488 laser line and RNAscope Probe-nCoV2019-orf1ab-sense-C2 (catalog number 859151) with Opal Dye 690 using the 640 laser line. After the RNAscope assay was completed, slides were washed in TBST [1× tris-buffered saline (TBS), pH 7.6, with 500 μl of Tween-20] for 2 min with agitation twice. Slides were incubated in the dark at room temperature for 30 min with 10% normal goat serum in 1× TBS with 1% bovine serum albumin (BSA). Blocking solution was removed from slides via flicking. Slides were then incubated in primary antibody solution using CD206 Antibody (clone C-10) AF546 from Santa Cruz Biotechnology (RRID:AB_10989352) at 1:100 dilutions in TBS 1% BSA for 1 hour at room temperature in the dark. Slides were rinsed using TBST for 5 min with agitation twice. Slides were then mounted and dried overnight. Images were taken in the Center for Advanced Microscopy at Northwestern University Feinberg School of Medicine using the Nikon W1-Spinning Disk Confocal microscope. Nucleus was added to the images using machine-based learning network trained on one patient using 4′,6-diamidino-2-phenylindole (DAPI) and bright-field images in Nikon Elements. Final images were rendered using Fiji.

### SHIELD fixation and imaging of lung tissue

The 100-μm lung slices were secondarily fixed using a derivation of the SHIELD fixation protocol (*33*). Slices were placed in clean 12-well tissue culture plates with 1 ml of SHIELD-Off solution (1:1:2 of ddH2O:SHIELD buffer:SHIELD epoxy). Plate was incubated at 4°C for 4.8 hours with agitation. Slices were transferred to a clean plate containing 1 ml of SHIELD-On solution (1:1 SHIELD-On buffer:SHIELD epoxy) and incubated for 2.4 hours at room temperature with agitation. Slices were cleared using passive methods, and slices were placed in a clean 12-well plate with LifeCanvas Passive Clearance Buffer at 37°C with agitation until slices were opaque; for healthy lung tissue, incubate for 30 to 60 min, and for diseased lung slices, incubate for about 4 hours. Slices were washed overnight in PBS with 1% Triton X-100 (PBST). Slices were stained in 1:10,000 dilution of Hoechst 33342 in PBST overnight. Slices were washed three times for 20 min in PBST after staining. Slices were placed in LifeCanvas Easy Index solution in a clean tissue culture plate for index matching.

The cleared tissue slices were imaged in the Center for Advanced Microscopy at Northwestern University Feinberg School of Medicine using the Nikon W1-Spinning Disk Confocal Microscope in a glass bottom dish at ×20 magnification. The 3D images were rendered using Fiji.

### Single-cell RNA-seq of lung tissue

Single-cell RNA-seq was performed using modifications to our published protocols (*14*). Distal lung biopsies were obtained from the explanted lung and donor lung from case 1, and the two postmortem biopsies. Two biopsies that included the main left bronchus and distal parenchyma from the upper lobe were obtained from another donor lung that was not placed for transplant (donor 1). A single biopsy from a distal lung parenchyma (donor 2) was obtained from wedge resection of the donor lung for size reduction during lung transplantation. Lung and airway tissues were infused with a solution of Collagenase D (2 mg/ml) and deoxyribonuclease I (0.1 mg/ml) in RPMI, cut into ~2-mm pieces, and incubated in 10 ml of digestion buffer with mild agitation for 30 min. The resulting single-cell suspension was filtered through a 70-μm nylon mesh filter, and digestion was stopped by addition of 10 ml of PBS supplemented with 0.5% BSA and 2 nM EDTA (staining buffer). Cells were pelleted by centrifugation at 300 rcf for 10 min, supernatant was removed, and erythrocytes were lysed using 5 ml of 1× Pharm Lyse solution (BD Pharmingen) for 3 min. The single-cell suspension was resuspended in Fc-Block (Human TruStain FcX, BioLegend) and incubated with CD31 microbeads (Miltenyi Biosciences, 130-091-935), and the positive fraction, containing endothelial cells and macrophages, was collected. The negative fraction was then resuspended in staining buffer, the volume was adjusted so the concentration of cells was always less than 5 × 10^7^ cells/ml, and the fluorophore-conjugated antibody cocktail was added in 1:1 ratio (EpCAM, Clone 9C4, PE-Cy7, BioLegend, catalog number 324222, RRID:AB_2561506, 1:40; CD206, Clone 19.2, PE, Thermo Fisher Scientific, catalog number 12-2069-42, RRID:AB_10804655, 1:40; CD31, Clone WM59, APC, BioLegend, catalog number 303116, RRID: AB_1877151, 1:40; CD45 Clone HI30, APCCy7, BioLegend, catalog number 304014, RRID: AB_314402, 1:40; HLA-DR, Clone LN3, eFluor450, Thermo Fisher Scientific, catalog number 48-9956-42, RRID:AB_10718248, 1:40). After incubation at 4°C for 30 min, cells were washed with 5 ml of MACS buffer, pelleted by centrifugation, and resuspended in 500 μl of MACS buffer + 2 μl of SYTOX Green viability dye (Thermo Fisher Scientific). Cells were sorted on a FACSAria III SORP instrument using a 100-μm nozzle and 20 psi pressure. Macrophages were sorted as live/CD45^+^HLA-DR^+^CD206^+^ cells, epithelial cells were sorted as live/CD45^−^CD31^−^EpCAM^+^, and stromal cells were sorted as live/CD45^−^CD31^−^EpCAM^−^ cells. Sample processing was performed in BSL-2 conditions using BSL-3 practices. Cells were sorted into 2% BSA in Dulbecco’s PBS (DPBS), pelleted by centrifugation at 300 rcf for 5 min at 4°C, and resuspended in 0.1% BSA in DPBS to ~1000 cells/μl concentration. Concentration was confirmed using K2 Cellometer (Nexcelom) with AO/PI reagent, and ~5000 to 10,000 cells were loaded on a 10x Genomics Chip B with Chromium Single Cell 3′ gel beads and reagents (3′ GEX V3, 10x Genomics). Libraries were prepared according to the manufacturer’s protocol (10x Genomics, CG000183_RevB). After quality check, single-cell RNA-seq libraries were pooled and sequenced on a HiSeq 4000 or NovaSeq 6000 instrument. Data were processed using Scanpy v1.5.1 (*34*), doublets were detected with scrublet v0.2.1 (*35*) and removed, samples were integrated with BBKNN v1.3.12 (*36*), cells were clustered with Leiden algorithm, and clusters were manually annotated.

To compare cell types in lung tissue biopsies from patients with COVID-19–induced lung fibrosis to those in lung tissue from patients with IPF, we obtained a dataset reported in Habermann *et al*. (*16*) using the accession number GSE135893. We then trained the scArches algorithm (*18*) on the Habermann *et al*. dataset and used it to integrate data from lung tissue of a patient with COVID-19. Briefly, scArches uses autoencoder neural networks to find a low-dimensional representation of the original dataset (reference) and performs transfer learning to project another dataset (query) into the same latent space. Despite being low dimensional, this latent space preserves transcriptomic variability of different cell types as well as heterogeneity within a given cell type. We used cell type annotations from Habermann *et al*. on our dataset to separate cells by lineage and performed clustering independently for cell lineages using latent space coordinates as input. The diagnosis metadata field was merged for both datasets and used for cluster composition analysis and for computing differentially expressed genes.

### Statistics

Patient demographics, postoperative complications, and outcomes were analyzed in the COVID-19 patient group who underwent lung transplantation. Continuous variables were reported as medians (interquartile means). Categorical variables were reported as number (percentage). Statistical analyses were performed using Stata/MP14 (StataCorp).

## SUPPLEMENTARY MATERIALS

stm.sciencemag.org/cgi/content/full/scitranslmed.abe4282/DC1 Fig. S1. Common histological features of warm lung autopsies. Fig. S2. Single-cell RNA-seq identifies similarities between end-stage pulmonary fibrosis and organizing pneumonia resulting from COVID-19. Table S1. Description of the specimens used in this study. Table S2. Marker genes for epithelial cell, macrophage, and mesenchymal cell clusters. Table S3. Differentially expressed genes in COVID-19 and IPF lung samples for each epithelial cell cluster. Table S4. Differentially expressed genes in COVID-19 and IPF lung samples for each macrophage cluster. Table S5. Differentially expressed genes in COVID-19 and IPF lung samples for each mesenchymal cell cluster. Movie S1. Three-dimensional matrix imaging of COVID-19 and non–COVID-19 lung tissue.

## Appendix

View/request a protocol for this paper from *Bio-protocol*.
